# FAMILY CAREGIVERS’ ADVANCE CARE PLANNING FOR COGNITIVELY IMPAIRED OLDER ADULTS: SERVICE PROVIDERS’ PERSPECTIVES

**DOI:** 10.1093/geroni/igz038.884

**Published:** 2019-11-08

**Authors:** Hyunjin Noh, Temilade A Aladeokin

**Affiliations:** The University of Alabama School of Social Work, Tuscaloosa, Alabama, United States

## Abstract

An increasing number of family caregivers face challenges of advance care planning (ACP) for their cognitively impaired older adults. The purpose of this study was to understand service providers’ views of ACP knowledge and needs among such family caregivers. Purposive sampling was used to recruit 10 service providers who serve older adults and their family caregivers in community settings of West Alabama. Individual, face-to-face interviews were conducted guided by a semi-structured questionnaire, asking about their experiences with and views of family caregivers’ ACP for their older adults. Thematic analysis of the qualitative data revealed several findings: family caregivers’ lack of knowledge about ACP and end-of-life care, discomfort in end-of-life discussions, uncertainty about their older adult’s end-of-life preferences, frustration with the surrogate decision-making role, family conflicts in ACP process, and logistical barriers to access ACP resources. Tailored services should be developed to address these barriers to promote ACP among this population.

